# Three years progress chronic limb-threatening ischemia case with simultaneous surgery

**DOI:** 10.1016/j.ijscr.2022.107212

**Published:** 2022-05-18

**Authors:** Yuta Terabe, Nobuhito Kaneko, Hiroshi Ando

**Affiliations:** Limb Salvage Center, Kasukabe Chuo General Hospital, 344-0063 midori5-9-4, Kasukabe, Saitama, Japan

**Keywords:** ABI, ankle brachial index, ADL, activities of daily living, CLTI, chronic limb-threatening ischemia, DFU, diabetic foot ulcer, EVT, endovascular therapy, HAV, hallux valgus, IPJ, interphalangeal joint, SFA, superficial femoral artery, SPP, skin perfusion index, Surgical offloading, Chronic limb-threatening ischemia, Hard-to-heal foot ulcer, Simultaneous surgery

## Abstract

**Introduction and importance:**

Chronic limb-threatening ischemia (CLTI) is a severe limb problem. The causes of foot ulcer are influenced by several factors, which lead to ischemia and foot deformity causing recurrence after wound healing. This report focuses on the long-term course.

**Case presentation:**

An 80-year-old male with CLTI was treated. The patient had an ulcer in the second toe of his left foot and stenosis of above the knee vessels. He performed his daily activities independently and his left foot was hallux valgus. After improving blood flow, foot deformity was corrected with ulcer treatment. The patient's second toe was amputated after the endovascular treatment. Finally, the toe was closed and hallux abductive valgus was corrected at the same time. After three years, he had no recurrence of foot ulcer. The patient uses a foot orthosis and his life is independent with no recurrence.

**Clinical discussion:**

Surgical offloading is an effective method to prevent recurrence. Surgical offloading is sometimes performed in patients with CLTI, but there is few occasion to undergo. This is because, there are problems of re-ischemia and an advanced age. Therefore, simultaneous surgery, as in this case, could be useful and help reduce the ulcer recurrence rate.

**Conclusion:**

Simultaneous surgery for CLTI was useful in leading to a reduction in recurrence rate.

## Highlights

• Simultaneous surgery (surgical offloading and ulcer closed operation) could be useful and help reduce the ulcer recurrence rate, because chronic limb-threatening ischemia has a problem of recurrence.
• After improvement of blood flow, chronic limb-threatening ischemia patient can be underwent surgical offloading.
• Patients who have undergone simultaneous surgery can maintain a long-term quality of life.
• Simultaneous surgery is relatively easy and saves surgeons time and effort to perfume surgical offloading another time.

## 1 Introduction

Chronic limb-threatening ischemia (CLTI) is a severe limb clinical syndrome associated with a high risk of limb amputation [1]. Several factors may elicit foot ulcers and the majority lead to ischemia and foot deformity due to neuropathy or non-neuropathy [2], and consequently exhibit recurrence after wound healing [3]. After improvement of blood flow, foot deformity is corrected with ulcer closed operation (simultaneous surgery). Simultaneous surgery produces preventing recurrence foot ulcer as well as wound healing. CLTI have a risk of ischemia again, hence simultaneous surgery is useful to perform while blood flow improves. The patients devote the rest period of the closed operation to the recovery from surgery to correct the foot deformity. However, increasing invasiveness is the risk of complications. Therefore, preoperative examination is important.

The long-term course case following simultaneous surgery is reported. The patient signed informed consents whenever they have treatment. This work has been reported in line with the SCARE criteria [4]. Written informed consent was obtained from the patient for publication of this case report and accompanying images. A copy of the written consent is available for review by the Editor-in-Chief of this journal on request. This work was registered in UMIN-CTR (https://www.umin.ac.jp/ctr/index-j.htm).

## 2 Case presentation

**Fig. 1:**
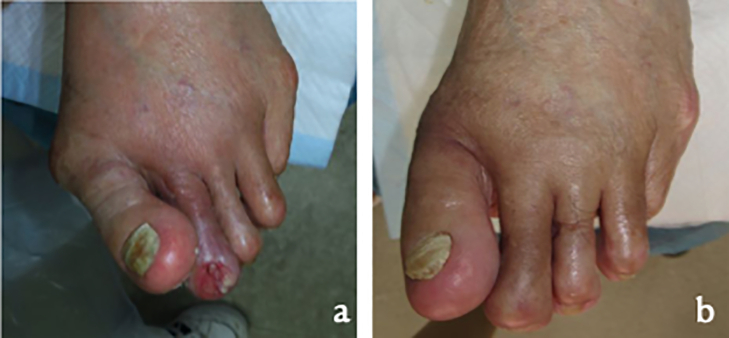
Ulcer in the second toe (a) and after three years of operation (b).

An 80-year-old male with CLTI and ulcer in the second toe of left foot presented to our hospital. The second interphalangeal joint (IPJ) was exposed (Fig. 1a). The second toe was mallet toe and hallux valgus (HAV) caused deformation of the toe (Fig. 3a). Additionally, patient had a stenotic superficial femoral artery (SFA), diabetes mellitus (HbA1c 7.3%), and cardiovascular disease. Ankle brachial index (ABI) was 0.90/0.52 (Rt./Lt.), skin perfusion index (SPP) was 31/83, 28/36 (mmHg) (Rt. dorsal/plantar, Lt. dorsal/plantar), and angiography exhibited chronic total occlusion of the left SFA. WIfI classification was W2, I2, fI1, F0.

**Fig. 2:**
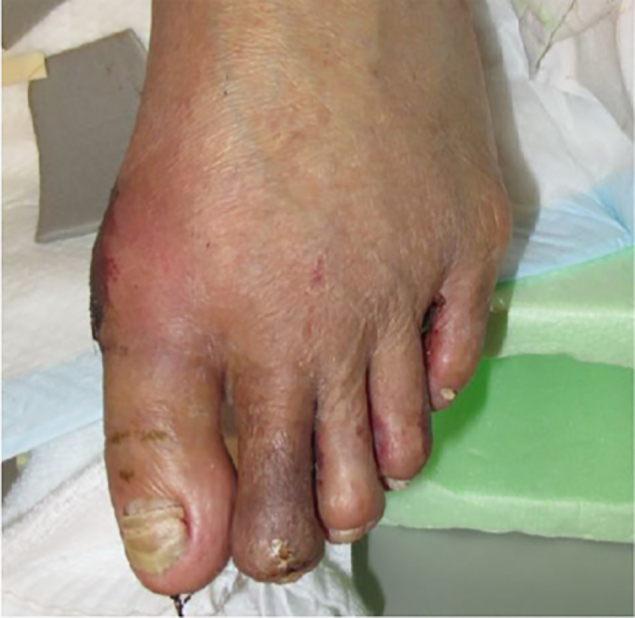
Post-operation (stump plasty of second ray and first ray distal osteotomy).

Endovascular therapy (EVT) was performed and stent implanted in the SFA region. ABI was 0.92/0.78 (Rt./Lt.) and SPP was 40/44 (Lt. dorsal/plantar) after EVT. The patient's second IPJ was excised after EVT. Osteomyelitis was improved and granulation grew. Finally, his second toe was closed and HAV was corrected with a simultaneous operation (Fig. 2).

**Fig. 3:**
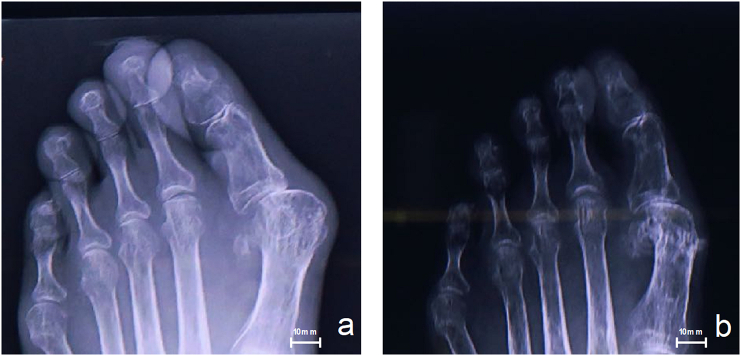
X-ray (a: pre operation, b: after three years of operation).

SFA re-occluded and EVT was performed only once, two years after the wound cured. After three years, the patient exhibited no recurrence of foot ulcer. He uses foot orthosis and lives an independent life (Figs. 1b, 3b).

## 3 Discussion

Simultaneous surgery of diabetic foot ulcer (DFU) case has ever reported [5], otherwise simultaneous surgery of CLTI case have never reported. Therefore, the case required performing EVT, treating wound, and correcting the foot deformity. Surgical offloading can be performed after improving blood flow. In this case, the effect of revascularization could be maintained for a long time because the EVT targets the SFA lesion [6]. Therefore, we were able to secure a period during which surgical offloading could be performed. In particular, there are few chances to perform surgical offloading after the wound is cured because CLTI patients are elderly and they always have risk of ischemia again.

In this surgical offloading, correction of the HAV, which was the cause of deformation of the second toe, was adjusted, and the stump formation of the second toe was able to match the length of the first toe. It is thought that the surgical offloading led to the prevention of wound recurrence. Due to CLTI, improvement of ischemia was needed. Since the lesion was above the knee this time, it was possible to expect patency for about a year by improving blood flow, which led to the simultaneous operation. The IWGDF guidelines also recommend surgical offloading for DFU, but not for CLTI [7]. CLTI have ischemia whether it's severe or not, therefore the patients are taken revascularization. So the patients are able to take the surgical offloading. However CLTI is at risk of ischemia again. Surgical offloading needs to be undergone before become ischemia again. As a result, simultaneous surgery is useful method.

CLTI and DFU patients performed major amputation have a low activites of daily living (ADL), and their mortality rate are also high [8], [9]. In addition, the adherence of DFU and CLTI is not so high, hence their recurrence rate is high [10], [11]. Recently, treatment results of CLTI have gradually improved [12]. Therefore, how to reduce the recurrence rate is important, and it is considered meaningful to reduce the recurrence rate by simultaneous surgery.

## 4 Conclusion

Simultaneous surgery for CLTI, performed at the same time as foot-ulcer treatment, was useful in leading to a reduction in recurrence rate. Preventing ulcer recurrence and maintaining ADL with simultaneous surgery is important for CLTI treatment.

## Sources of funding

None.

## Ethical approval

This study was approved by the Research Ethics Committee of our Hospital (Permission No. 2103-1).

## Consent

Written informed consent was obtained from the patient for publication of this case report and accompanying images. A copy of the written consent is available for review by the Editor-in-Chief of this journal on request.

## CRediT authorship contribution statement

Conception and design of study: Yuta Terabe,

Acquisition of data: Yuta Terabe, Nobuhito Kaneko.

Analysis and interpretation of data: Yuta Terabe.

Drafting the manuscript: Yuta Terabe.

All authors contributed in writing the paper.

## Research registration

UMIN Clinical Trials Registry (No.R000053878, ID: UMIN000047239), https://www.umin.ac.jp/ctr/index-j.htm

## Guarantor

Yuta Terabe

## Provenance and peer review

Not commissioned, externally peer-reviewed.

## Declaration of competing interest

None.
